# Clinical Aspects associated with Syndromic forms of Orofacial Clefts in a Colombian population

**Published:** 2015-12-30

**Authors:** Liliana Arias Urueña, Ignacio Briceño Balcazar, Julio Martinez Lozano, Andrew Collins, Daniel Alfredo Uricoechea Patiño

**Affiliations:** 1 Medical School. Universidad de La Sabana, Bogota, Colombia; 2 Pontificia Universidad Javeriana. Bogota, Colombia; 3 Genetic Epidemiology and Bioinformatics. University of Southampton. Southampton, UK

**Keywords:** Cleft lip and palate, Pre-eclampsia, Aarskog-Scott syndrome, Congenital Hip Dysplasia

## Abstract

**Objectives:**

To present descriptive epidemiology of Orofacial Clefts and to determine the association of syndromic forms with antenatal high-risk conditions, preterm birth, and comorbidities among nested-series of cases.

**Methods:**

A study of nested-series of cases was conducted. Frequencies of cleft type, associated congenital anomalies, syndromic, non-syndromic and multiple malformation forms, and distribution of Orofacial Clefts according to sex and affected-side were determined. Odds ratios were calculated as measures of association between syndromic forms and antenatal high-risk conditions, preterm birth and comorbidities. A total of three hundred and eleven patients with Orofacial Clefts were assessed in a 12-month period.

**Results:**

The most frequent type of Orofacial Clefts was cleft lip and palate, this type of cleft was more frequent in males, whereas cleft palate occurred more often in females. The most common cases occurred as non-syndromic forms. Aarskog-Scott syndrome showed the highest frequency amongst syndromic forms. Hypertensive disorders in pregnancy, developmental dysplasia of the hip, central nervous diseases and respiratory failure showed significant statistical associations (*p* <0.05) with syndromic forms.

**Conclusions:**

These data provide an epidemiological reference of Orofacial Clefts in Colombia. Novel associations between syndromic forms and clinical variables are determined. In order to investigate causality relationships between these variables further studies must be carried out.

## Abstract (es)

**Objetivos:**

Presentar la epidemiología descriptiva en torno a las Fisuras Orofaciales y determinar asociaciones entre Fisuras Orofaciales sindromica y antecedentes antenatales de alto riesgo, parto pretérmino, y comorbilidades en una población Colombiana.

**Métodos:**

Se planteó un estudio de serie de casos anidado estratificado. Se calcularon frecuencias en relación al tipo de fisura desde el punto de vista anatómico, anomalías congénitas paralelas, morbilidades y forma clínica. Se analizó la distribución de las Fisuras Orofaciales de acuerdo al género y lateralidad. Se determinaron razones de disparidad entre la forma sindrómica y antecedentes antenatales de alto riesgo, parto pretérmino, y comorbilidades. Se evaluaron trecientos once pacientes que asistieron a la consulta de genética clinica durante un año.

**Resultados:**

La Fisura Labio-palatina fue el tipo más frecuente en la muestra evaluada y la más frecuente en hombres. La Fisura Palatina fué la más frecuente en mujeres, la forma clínica más común fue la no sindrómica. En la población sindrómica el Síndrome de Aarskog-Scott mostró la frecuencia más alta. Los trastornos Hipertensivos de Embarazo, la Displasia del Desarrollo de la Cadera, las enfermedades respiratorias y del sistema nervioso central mostraron una asociación estadísticamente significativa con la forma sindrómica. (*p *<0.05).

**Conclusiones:**

Estos datos ofrecen una referencia epidemiológica descriptiva de las Fisuras Orofaciales en Colombia. Las asociaciones encontradas entre los aspectos clínicos estudiados y la forma sindrómica, deben ser investigadas en próximos estudios con el fin de determinar relaciones de causalidad.

## Introduction

Orofacial clefts (OFC) represent one of the most common birth defects, occurring frequently in Asians and Amerindians 1-3. Affected subjects tend to have language and hearing problems and difficulty in social integration, therefore multidisciplinary care is required in order to improve health status 4. 

Based on their association with specific malformative patterns or their presence as isolated defects, OFCs can be classified as syndromic (SF) and nonsyndromic form (NSF), respectively 5. Approximately 30% of cases of Cleft Lip and Palate (CLP) occur as SF 6,7. Patients affected by SF tend to have higher morbidity and mortality throughout life due to their associated congenital anomalies 4. Given the complex etiology and pathogenesis of these anomalies, patients need genetic assessment to establish an accurate diagnosis and appropriate risk management 8. 

The prevalence of OFCs depends largely on factors such as ethnicity and geographic region 9. Frequently, facial clefts are associated with other congenital defects 4,10. The study of past medical and family history and associated anomalies is useful in understanding inheritance patterns, risk factors and in providing public health strategies 8. 

No research in Colombia has addressed a complete descriptive epidemiology of OFC or the relationship of OFC with some clinical aspects 11,12, therefore providing epidemiological information is a research priority area. The current study was designed to: 1) present the frequency of cleft type, associated congenital anomalies, syndromic, non-syndromic and multiple malformation forms; 2) determinate associations between syndromic forms and antenatal high- risk conditions, preterm birth and comorbidities. 

## Materials and Methods

### Subjects 

Three hundred and eleven individuals with Orofacial Clefts aged between 3 weeks and 52 yrs who attended at Operation Smile Colombia from April 2012 to July 2013 were assessed by Medical Genetics Team at Operation Smile Colombia. A recruiting was not performed. The whole population was included in this study. Sampling was not carried out. 168 (52%) were males, 149 (48%) were females. Distribution by age is shown in Table 1. Ethical principles for medical research involving human subjects, as outlined in the declaration of Helsinki were followed. Universidad de La Sabana ethical committee approved the study protocol. 

**Table 1.** Sex, age and region of origin (N= 311).

Variable		n	%
Sex	Male	168	54
	Female	149	48
Age range (yrs)	<1 m	7	2
	<1	105	34
	2-5	41	13
	6-11	50	16
	12-17	58	19
	≥18	50	16
Origin area	Rural	137	44
	Urban	174	56

m= month

### Procedure

Information about sex, type of cleft, past medical and family history was recorded in children (<18 yrs) and adults (≥18 yrs). In children, maternal, and pediatric history were recorded focusing on antenatal high-risk conditions, the presence or absence of preterm birth, comorbidities and neonatal diseases. Pregnancy dietary supplements and /or folate intakes were not assessed. Preterm birth was defined as delivery at ≤37 weeks gestation. Two trained physicians in clinical genetics performed a physical examination focusing on identifying other congenital anomalies and establishing a clinical diagnosis. 

Based on clinical features the patients were classified into 3 categories:

1. Non- syndromic form (NSF): patients affected by isolated OFCs.

2. Syndromic form (SF): patients affected by OFCs and a specific syndrome can be recognized (OMIM). 

3. Multiple malformation form (MMF): patients affected by OFCs and other malformations but a specific syndrome cannot be recognized.

4. A whole-exome sequencing was used to resolve clinical diagnoses for some syndromic phenotypes.

### Data analysis 

Cross tabulation was used to analyze the frequency distribution of the variables (sex, age, region of origin, cleft type, affected-side, clinical form, associate anomalies, morbidities). In order to determinate a measure of association between the occurrences of interest (antenatal high-risk conditions, presence or absence of preterm birth, and comorbidities) and SF of OFC, two cases were defined. Case 1: cases with SF (224). Case 2: cases with NSF (59). Taking into account MMF does not have any specific pattern it was not included in any case group. 

Chi-square statistics, Fisher´s exact test and odds ratio (OR) calculations were used to determine associations. The frequency of the ocurrences in SF group to NSF group was compared. Results were considered to be significant at *p *<0.05. All data were analyzed using Epi Info version 7^®^ 13. 

## Results

The most common sex, age range and region of origin were male, 1-23 months and urban area respectively (Table 1). The most frequent type of OFC was CLP (69%). Analysis of cleft type by sex showed that CLP was more frequent in males, whereas Cleft Palate (CP) occurred more often in females (Table 2). The majority of CLP cases were left-sided (55.3%). Seventy two percentage of cases occurred as NSF, and 20% had a recognized-syndrome (Table 3). The most frequently identified syndromes were Aarskog-Scott and Velocardiofacial (Table 4). Among the 288 (92.6%) of patients who had an additional congenital defect, musculoskeletal, cardiovascular, urogenital and nervous systems were the most common types. Among children 79.0% showed at least a morbidity (Table 3).

**Table 2.** Cleft type distribution according to sex.

Variable	Female	Male	Total
CL	13	7	20
CL±A	4	4	8
CLP	91	125	216
CP±A	1	3	4
CP	41	22	63
TOTAL	150	161	311

CL= cleft lip; CL±A= cleft lip with or without cleft alveolus; CLP= cleft lip and palate; CP±A= cleft palate with or without cleft alveolus; CP= cleft palate

**Table 3.** Frequency of clinical forms, congenital anomalies with orofacial clefts and morbidities in Children and Adults^∗^

Variables		n	%
Clinical forms	MMF	28	9.0
	NSF	224	72.0
	SF	59	19.0
	Total	311	
Birth according to clinical form in children			
Term birth	MMF	21	11.0
	NSF	137	70.0
	SF	37	19.0
	Total	195	
Preterm birth
	MMF	3	4.0
	NSF	48	73.0
	SF	15	23.0
	Total	66	
Morbidities			
Children (<18 yrs)	0	40	80.0
	1	5	10.0
	2	5	10.0
	≥3	0	
	Total	50	
Adults (18 ≥yrs)
	0	141	54.0
	1	65	25.0
	2	38	14.6
	≥3	17	6.4
	Total	261	
Associated congenital anomalies with orofacial clefts			
System or organ
	Nervous	27	8.7
	Eye	10	3.2
	Cardiovascular	28	9.0
	Urogenital	27	8.7
	Musculoskeletal	160	51.4
	Oral Cavity	12	3.9
	Intergument	24	7.7
	No	23	7.4
	Total	311	

^*^Birth history was asked among pediatric population. Birth history is not included within Adult Medical History. Adults were not included in this analysis.

MMF= Multiple malformation form; NSF= Non-syndromic form; SF= Syndromic form

**Table 4.** Frequency of syndromes associated to orofacial clefts

Code	Mendelian Inheritance in Man	n	%
305400	Aarskog-Scott	10	17.0
101200	Apert	1	1.7
601701	Arthrogryposis and Ectodermal Dysplasia	1	1.7
123500	Crouzon	1	1.7
305100	Ectodermal Dysplasia and Hypohidrotic 1	3	5.1
129900	Ectrodactily, Ectodermal Dysplasia and Cleft Lip Palate 1	1	1.7
129830	Ectrodactyly Cleft Palate	1	1.7
-	Fetal Alcohol	1	1.7
164210	Hemifacial Microsomia	1	1.7
601471	Heriditary Congenital Facial Paresis 1	1	1.7
142900	Holt-Oram	1	1.7
300337	Hypomelanosis of Ito	1	1.7
-	Klinefelter	1	1.7
154700	Marfan	2	3.4
163950	Noonan	1	1.7
6002510	Oblique Facial Clefting 1	1	1.7
311200	Orofaciodigital 1	3	5.1
133900	Orofaciodigital 5	1	1.7
304120	Otopalatodigital 2	2	3.4
261800	Pierre Robin	5	8.4
119500	Popliteal Pterygyum	2	3.4
106600	Selective Tooth Agenesis 1	1	1.7
117550	Sotos	2	3.4
-	Turner Syndrome	1	1.7
192350	VACTERL association	1	1.7
119300	Van der Woude 1	3	5.1
192430	Velocardiofacial	10	17.0
	Total	59	

Code= From OMIM, Catalog of Human Genes and Genetic Disorders

The distribution of preterm birth was similar among MMF, SF and NSF populations. (Table 5). The only antenatal high-risk condition that showed significant statistical association with SF was the spectrum of Hypertensive Disorders in Pregnancy (*p*= 0.05). Preterm birth did not show significant statistical association with SF (*p*= 0.67). Heart diseases, respiratory failure, seizures, and developmental dysplasia of the hip had significant statistical associations with SF (*p*= 0.000, *p*= 0.0005, *p*= 0.002, * p*= 0.0006, respectively) (Table 5) .

**Table 5.** Association of SSF and NSF with antenatal high-risk conditions in Children and comorbidities among children and adults.

Variables		SSF	NSF			
		n	n	Total	OR	p
Antenatal risk						
Preterm Labor	Yes	1	1	2	
	No	48	184	232	3.8	0.3700
Oligohydramnios	Yes	1	6	7		
	No	48	179	227	0.6	1.0000
HDP	Yes	7	11	18	
	No	42	174	216	2.6	0.0500
Bleeding (unknown cause)	Yes	1	5	6	
	No	48	180	228	0.7	1.0000
FGR	Yes	1	6	7	
	No	48	179	227	0.6	1.0000
Fetal distress	Yes	1	1	2	
	No	48	184	232	3.8	0.3700
PPRM	Yes	1	2	3	
	No	48	183	231	1.9	0.5000
Comorbidities						
Respiratory infectious	Yes	10	26	36	
	No	39	159	198	1.6	0.3000
Gastrointestinal Tract diseases	Yes	7	13	30	
	No	42	172	214	2.2	0.1000
Heart diseases	Yes	14	2	16	
	No	35	183	218	36.7	0.0000
DDH	Yes	6	3	9	
	No	43	182	225	8.5	0.0006
Respiratory Failure	Yes	7	7	14	
	No	42	178	220	4.2	0.0005
Diseases of the Newborn	Yes	6	16	22	
	No	43	169	212	1.5	0.4400
Ophthalmopathy	Yes	3	4	7	
	No	46	181	227	2.95	0.1500
CMO	Yes	4	27	31	
	No	45	158	203	0.5	0.2300
Seizures	Yes	8	5	13	
	No	41	180	221	7	0.0020
Kidney and urinary tract diseases	Yes	2	2	4	
	No	47	183	230	3.9	0.1500

SSF= syndromic form; NSF= nonsyndromic form; HDP= Hypertensive Disorders in Pregnancy; FGR= Fetal growth restriction; PPRM= Preterm premature rupture of membrane; DDH= developmental dysplasia of the hip; OR= odds ratio

## Discussion

The present work is the first complete epidemiological descriptive study about Orofacial Clefts in Colombia 11,12,14. Our results are consistent with previously published studies of the distribution of OFC according to sex, affected-side and cleft type 6,7,15-17. 

Aarskog-Scott syndrome (AAS) shows the highest frequency among SF. This observation differs from previously published papers, which reported Van der Woude Syndrome (VDW) as the most common 6,7,18. Aarskog-Scott syndrome is an X-linked condition caused by mutations of the *FGD1* gene. It is a clinically and genetically heterogeneous condition characterized by facial dysmorphic features, short stature, brachydactyly, and genital anomalies 19,20. Although clinical manifestations and diagnostic criteria are well established, diagnosis is not simple, due to the extremely variable spectrum of phenotypical features 21,22. It is probable that AAS is being underdiagnosed and for that reason the frequency according to previous studies appears lower. Further studies must be. 

However, geographical and ethnic factors of our population should be considered, given that they might influence the distribution of the SF with respect NSF. Research into *FGD1* founder mutations might be usefully conducted in future studies.

The musculoskeletal system is the most frequently affected among SF population according to this research. This result is consistent with reported findings by Calzolari *et al* 23. This may reflect the impact of a number of genes which play an essential role in the development of connective tissue 4,24. 

According to Sekhon *et al*^25^ facial anomalies are the most frequently detected, followed by ocular, central nervous system, lower and upper extremities and cardiovascular. Most of the facial, lower and upper extremities anomalies involve connective tissue. It is important to consider that the published prevalence of associated anomalies vary considerably depending on methodological factors 26. 

The roles of antenatal high-risk conditions among the SF population have not been well studied. Our work provides the first evidence that there is an association between SF and hypertensive disorders in pregnancy in comparison with NSF (OR= 8.5) . 

The etiology of SF is related to mutations within several genes involved in mesenchymal and epithelial proliferation, cell adhesion and migration and angiogenesis. All of these are essential for lip and palate development 7,27,28. The disturbance of decidua-trophoblast interactions during early human pregnancy is one of the events implicated into the pathogenesis of hypertensive disorders in pregnancy 29-31. These interactions depend largely on maternal uterine endothelial cells activated by expression of selectins that enable adherence of trophoblast to maternal endothelium 32,33, and epithelial-mesenchymal transition during trophoblast differentiation 34,35. Given the above we propose that common processes may be disrupted in both entities: 1) cell adhesion mechanisms, 2) epithelial-mesenchymal transition, and 3) angiogenesis. 

Transforming growth factor-beta 3 (*TGF-β3*), plays an essential role in these processes, and is known to be involved in the pathogenesis of hypertensive disorders in pregnancy 36-39 and some forms of OFCs 36,40. Therefore, it might be a candidate gene for both disorders. In order to test this hypothesis this gene should be investigated in patients and their mothers affected by SF and preeclampsia respectively. Associations of SF and developmental dysplasia of the hip (DDH) have not been reported in previous papers. The etiology of DDH is multifactorial, but has a considerable genetic component 41,42. Although oligohydramnios is a risk condition associated with DDH, the relationship between SF and oligohydramnios does not show significant statistical association according to this work. The causality relationships underlying this finding must be investigated with regard to the possibility of earlier hip screening among this population. 

Desalu *et al*. 43, reported that anatomical abnormalities associated with cleft lip and palate increase the risk of airway complications and this is confirmed by comparing SF and NSF in the current study (OR= 4.2). Clinical features such as micrognathia 44 and congenital heart diseases are common in SF; these factors might be involved in this association.

Preterm birth and other antenatal high-risk conditions do not show significant statistical association with SF, probably due to limited power given the small set of observations. 

The associations found in this study contribute to appropriate medical and risk management of the affected patients. Clinicians can be guided by this study in order to provide comprehensive care for the benefit of these patients and their families. Based on the findings of this work, we are performing molecular diagnosis of the SF cases. Establishing causality relationships between the studied variables is one of the central goals of our future studies. 

## Conclusion

 These data provide an epidemiological reference of Orofacial Clefts in Colombia. Novel associations between syndromic forms and clinical variables are determined. In order to investigate causality relationships between these variables further studies must be carried out. 
